# Retention of *Pantoea agglomerans* Sc1R across stadia of the southern green stink bug, *Nezara viridula* (L.) (Hemiptera: Pentatomidae)

**DOI:** 10.1371/journal.pone.0242988

**Published:** 2020-12-03

**Authors:** Jesus F. Esquivel, Enrique G. Medrano

**Affiliations:** Insect Control & Cotton Disease Research Unit, Plains Area, Agricultural Research Service, United States Department of Agriculture, College Station, Texas, United States of America; University of California Riverside, UNITED STATES

## Abstract

Southern green stink bug [*Nezara viridula* (L.)] adults and other pentatomid pests can transmit pathogens (e.g., the bacterium *Pantoea agglomerans*) that cause disease in cotton (*Gossypium hirsutum* L.) and other high-value cash crops worldwide. First instars of *N*. *viridula* were recently shown to ingest *P*. *agglomerans* strain Sc1R with rifampicin-resistance, and to retain the pathogen to the 2^nd^ instar. The objective of this study was to determine the acquisition of *P*. *agglomerans* Sc1R by early instars of *N*. *viridula* and determine persistence of *P*. *agglomerans* Sc1R across subsequent stadia. In three trials, early instars (1^st^ and 2^nd^) were exposed to *P*. *agglomerans* Sc1R and subsequently maintained to adulthood; cohorts were sampled at 3^rd^ and 5^th^ instars, as well as adults. In every trial, *P*. *agglomerans* Sc1R was detected in all stadia, including adults, but significantly higher frequencies of infection than expected were observed at the initial stage of infection (either 1^st^ or 2^nd^ instar). Higher densities of *P*. *agglomerans* Sc1R were detected in 1^st^ and 2^nd^ instars, and lower densities were observed in subsequent stadia. Densities of innate microbiota were generally lower when the initial stage of exposure was at 1^st^ instar than when the initial stage of exposure was at the 2^nd^ instar. Overall, half of the adults possessed *P*. *agglomerans* Sc1R. These findings demonstrated that *N*. *viridula* nymphs can acquire *P*. *agglomerans* Sc1R and retain the pathogen to adulthood. Potential avenues of research to further elucidate the implications of nymphs harboring pathogens to adulthood are discussed.

## Introduction

Stink bugs (Hemiptera: Pentatomidae) continue to plague cotton (*Gossypium hirsutum* L.) production in the Cotton Belt of the U.S. and worldwide. In the U.S., this persistent threat can be partially attributed to a result of reduced insecticide applications prompted by successful boll weevil (*Anthonomus grandis grandis* Boheman; Coleoptera: Curculionidae) eradication and the virtually complete adoption of cotton plants containing the Bt (i.e., *Bacillus thuringiensis* Berliner) toxin for control of lepidopterous pests. Also, stink bugs are extremely polyphagous, feeding on both cultivated and uncultivated plants. For example, the southern green stink bug [*Nezara viridula* (L.)] has been associated with feeding upon, or utilizing, 43 plant families, comprised of 197 plant taxa distributed within 39 genera and 158 species [1]. The elevated pest status is further complicated because adults of southern green stink bug (*N*. *viridula*) and brown stink bug (*Euschistus servus* Say) are known to ingest and transmit *Pantoea agglomerans* (Ewing and Fife) [2–4], a causal organism of seed- and boll-rot in cotton [5]. Afflicted cotton results in decreased quality and quantity in seed and lint, thereby affecting a producer’s profit margin.

Persistent pathogen infection within an individual stink bug amplifies the threat of pathogen transmission by stink bugs in cotton and other row crops. For example, individual adult southern green stink bugs infected with a fungal pathogen [i.e., *Eremothecium coryli* (Peglion) Kurtzman (syn. *Nematospora coryli* Peglion) [6]] successfully inoculated multiple consecutive cotton bolls [7]. Specifically, bolls exposed early (i.e., 1^st^ boll) within a sequence of 5 bolls expressed similar densities of *E*. *coryli* as those densities found in the last bolls exposed (i.e., 5^th^ boll) to infected adults throughout a 15-day study period. These findings indicated that, once introduced into a boll, *E*. *coryli* proliferated at a similar rate in each boll, resulting in disease. Further, individual adults remained infected with *E*. *coryli* at the conclusion of the 15-day study period [7], demonstrating that adult southern green stink bugs can retain *E*. *coryli* for ≥15 d after pathogen acquisition.

Persistence of plant pathogens across some stink bug stadia is documented [8, 9]. First instars of *N*. *viridula* were observed feeding and ingesting a marked bacterial pathogen (i.e., *Pantoea agglomerans* Sc1R), subsequently retaining the pathogen to the 2^nd^ instar [9]. This prompted the question whether pathogens ingested by early instars could be retained to adulthood and, if so, whether temporal pathogen densities varied across stadia. The objective of this study was to determine the acquisition of *P*. *agglomerans* Sc1R by early instars of *N*. *viridula* and determine persistence of *P*. *agglomerans* Sc1R across subsequent stadia. Indeed, adults of southern green stink bug were shown to harbor *P*. *agglomerans* Sc1R.

## Materials and methods

### Insect source

A colony of *N*. *viridula*, comprised of a combination of laboratory adult insects obtained from USDA, ARS (Stoneville, MS, USA) and supplemented with field-collected adults near the study site, was maintained on rinsed fresh green beans (*Phaseolus vulgaris* L.) and corn (*Zea mays* L.). All adults were held in BugDorm-1 containers (Mod. No. 1452, 299 cm^3^; BioQuip Products, Rancho Dominguez, CA, USA) and provisioned with two whole ears of shucked corn (i.e., husks completely removed) and approximately 12 pods of green beans per container. The food items were replaced twice per week at 3–4 d intervals. Also, at each food replacement, available egg clutches were harvested and placed in modified plastic containers as previously described [10], Esquivel & Medrano (2012). Nymphs resulting from these egg clutches and colony insects were held in a walk-in environmental chamber at 26.7±1.0 °C and 14:10 [L:D] photoperiod.

### Preparation of pathogen for exposure to insects

Established methodologies [3, 5] were followed to expose the insects to the rifampicin-resistant strain of *P*. *agglomerans* Sc1R (described below). Pathogen cultures stored for 3 years in 30% glycerol and held at -20 °C were used to acquire single colonies of the bacterium. Isolates were routinely grown on Luria Bertani agar (LBA; Difco Laboratories, Detroit, MI, USA) amended with rifampicin (LBAR; 100 μg/ml) at 27 °C. A remote colony selected for experiments was retested for cotton boll virulence as previously described [5].

Fresh, intact green bean pods were sterilized by subjecting batches of ca. 15–25 pods to 121 °C for 20 min at 1 kg cm^-2^ pressure using a sterilizer (Consolidate Sterilizer Systems, Boston, MA, USA). Bean sterility was verified using the quality control methods previously described [2, 3, 5]. A sterile scalpel was used to cross-section the pods into ≈3-cm sections; bean ends were discarded.

A bacterial suspension prepared from 18-h cultures in sterile water was adjusted spectrophotometrically (A_600_ = 1.0) resulting in 10^11^ cells ml^-1^. The inoculum for the green beans consisted of 1-ml of the pathogen suspension diluted with 49 ml of sterile water. Green bean sections were soaked for 2 min in either the pathogen inoculum or sterile water. After removing the bean sections from the pathogen suspension or sterile water, bean sections were blotted dry with sterile paper towels and aseptically placed in a sterile disposable Petri dish (100 × 15 mm). Insects were exposed to either the sterile beans (i.e., non-treated control) or beans infected with *P*. *agglomerans* Sc1R as described below.

### Exposure of *P*. *agglomerans* Sc1R to insects

Three independent trials were performed to assess ingestion of *P*. *agglomerans* Sc1R by early instars and retention across various stadia. The first trial was initiated at 1^st^ instar but a higher mortality rate than expected occurred, presumably due to handling of the fragile neonate 1^st^ instars; thus, exposure to treatments in the subsequent two trials were initiated at the 2^nd^ instars.

For each trial, 25 individual egg clutches nearing hatch (as indicated by the red eyes visible through the dorsal egg chorion) were placed in individual sterile disposable Petri dishes (100 × 15 mm). Beginning with 1^st^ instars (trial 1), nymphs were fed for 2-d either sterile beans or beans infected with *P*. *agglomerans* Sc1R; nymphs hatching from 20 clutches received beans infected with *P*. *agglomerans* Sc1R and the remaining 5 clutches were provided the sterile beans (as a control treatment). For trials 2 and 3, the nymphs were exposed to *P*. *agglomerans* Sc1R at the 2^nd^ instar, but the baseline sample sizes were as described immediately below for trial 1 with the exception that the baseline samples were taken at 2^nd^ instar. In these latter trials (trials 2 and 3), however, 1^st^ instars were provisioned with non-sterile fresh green beans (previously rinsed in water) given that 1^st^ instars will feed when provided a food source [9]. All nymphs used in this study were held in a walk-in environmental chamber at 26.7±1.0 °C and 14:10 [L:D] photoperiod.

After the initial 2-d exposure period and collection of baseline samples, remaining study insects were placed in individual Petri plates and provided a ≈3-cm section of rinsed green bean (i.e., non-autoclaved/non-sterile) until each insect reached adulthood. The green bean sections were replaced on Monday, Wednesday, and Friday (using new Petri plates at each feeding).

### Detection of *P*. *agglomerans* Sc1R in insects

#### Baseline samples for *P*. *agglomerans* Sc1R and innate bacteria

At the conclusion of the 2-d feeding period, baseline samples for trial 1 were collected and processed (as described below at **Microbiological assays**) to determine the presence of *P*. *agglomerans* Sc1R in cohorts of 1^st^ instars from each treatment. In total, for trial 1, 20 samples of 1^st^ instar nymphs (i.e., 20 tubes with five 1^st^ instars per tube) and 5 samples of 1^st^ instars (i.e., 5 tubes with five 1^st^ instars per tube) served as a relative baseline to assess concentrations of *P*. *agglomerans* Sc1R and innate microbiota, respectively. Baseline samples for trials 2 and 3 were as described for trial 1 with exception that 2^nd^ instars instead of 1^st^ instars were examined given that 2^nd^ instar was the stage of initial exposure for trials 2 and 3.

#### Temporal samples for *P*. *agglomerans* Sc1R and innate bacteria across stadia

To examine retention of *P*. *agglomerans* Sc1R across stadia, insects were collected at the 3^rd^ and 5^th^ instar (*n* = ≥20 nymphs per stadium). In contrast to using cohorts of five insects per sample (i.e., as in baseline sampling), each insect was processed individually (see below). Remaining insects continued to receive green bean sections as previously described until each insect reached adulthood. All insects that survived to adulthood were examined individually to determine the presence and densities of *P*. *agglomerans* Sc1R. Individual samples of 3^rd^ instars, 5^th^ instars, and adults were individually placed in a sterilized container and pulverized to yield microbial suspensions for each sample, as described below.

#### Microbiological assays

The process to assess the acquisition of the rifampicin resistant Sc1R strain and enumerate normal microbiota began with surface sterilization of insect samples. For each baseline sample and subsequent temporal samples, 5 ml of 70% ethanol was added to each 15 ml tube and incubated for 5 min. The samples were then rinsed by transferring them to tubes that contained 5 ml of sterile water for 5 min. Each sample was transferred into a 1.1 ml microtube (in strips of eight tubes) that contained both a sterile 4-mm stainless steel ball (SPEX SamplePrep, Metuchen, NJ, USA) and 0.5 ml of sterile water. For pulverization, the strips were placed in a rack of 96 tubes. The grinding parameters consisted of agitation for 5 min at 1500 strokes per min using 2000 Geno/Grinder (SPEX SamplePrep) to yield homogenate suspensions.

To determine the presence of *P*. *agglomerans* Sc1R and normal bacterial fauna, homogenate suspensions from each sample were 10-fold dilution plated using sterile water on both LBA and LBAR. The non-selective LBA medium was used to detect any culturable innate bacterial fauna in the samples; the amended LBAR medium detected the rifampicin-resistant *P*. *agglomerans* Sc1R strain. After two days of incubation at 27° C, colony forming units (cfus) of *P*. *agglomerans* Sc1R and innate bacteria were enumerated and recorded as cfu/g tissue for each stadium.

### Statistical analyses

Although 1^st^ instars have been shown to feed [9], significant mortality was observed during trial 1 thereby prompting modification to initiate subsequent trials using 2^nd^ instars. The cause of mortality was presumably due to handling of the newly hatched insects. Since trial 1 was initiated with 1^st^ instars and trials 2 and 3 were initiated with 2^nd^ instars, trial 1 was analyzed separately and observations for trials 2 and 3 were pooled.

#### Frequency of *P*. *agglomerans* Sc1R infection across stadia

The PROC FREQ procedure [11] was used for contingency table analyses to compare disease presence (i.e., infection) in each of the stadia for trial 1 and pooled trials 2 and 3. For these comparisons, disease presence was in columns and stadia was in rows. The EXPECTED option was invoked to generate the expected number of infections (i.e., observations) for comparison with the observed number of infections. The EXACT option was invoked to generate the Fisher’s Exact statistic to determine significance of the association between the observed and expected frequencies of infection.

#### Densities of *P*. *agglomerans* Sc1R across stadia

The limitations of the bioassay for detecting cfus affected lower and upper densities of cfus. Specifically, the minimum cfu threshold of detection for the bioassay was 10^1^ cfus but it is plausible that *P*. *agglomerans* Sc1R could have been present at densities less than 10^1^ cfus. To account for the limitations of the bioassay, the density data was categorized as <10^1^ and assigned a value of 0.1 cfus instead of a zero value for analyses. Similarly, the maximum threshold of detection was 10^9^ and, where cfus exceeded this limit, the density data was categorized as >10^9^ and instead assigned a value of 1,000,000,000 cfus for log transformations and analyses.

To mitigate bias posed by the raw density data (cfus) for general microbiota and *P*. *agglomerans* Sc1R, the raw data were log-transformed to fit the assumption of normal distribution for an analysis of variance. The PROC MIXED procedure was invoked to generate the analysis of variance [11]. For trial 1 (i.e., 1^st^ instars as stage of exposure to *P*. *agglomerans* Sc1R), the CLASS variables were stadium and sample number; the MODEL statement included the log-transformed density of *P*. *agglomerans* Sc1R as the dependent (response) variable and the insect stadium as the independent variable. The sample number was the RANDOM variable. Finally, the LSMEANS statement was invoked to generate mean log-transformed densities (i.e., LSmeans) and the ADJUST = TUKEY option was used to generate the Tukey-Kramer Adjusted *P* statistic for comparison of LSmeans estimates between stadia. Back-transformed means and range of back-transformed means for densities of *P*. *agglomerans* Sc1R, based on LSMeans estimates, were generated in Excel using the following formulae: means = 10^estimate; range, = 10^estimate+SE (for upper boundary) and 10^estimate-SE (for lower boundary). To assess the log-transformed densities of general bacteria, the syntax was as described immediately above except that the log-transformed density of general bacteria was the dependent (response) variable. Methods for generating back-transformed means and range of back-transformed means were as described above for determination of similar data for *P*. *agglomerans* Sc1R.

When trials 2 and 3 were examined separately, post-hoc preliminary examination of covariance parameter estimates indicated the trials could be pooled. As mentioned above (see **Statistical Analysis**), these two trials were initiated when 2^nd^ instars were exposed to *P*. *agglomerans* Sc1R, unlike trial 1 where 1^st^ instars were used. Similar to trial 1, the PROC MIXED procedure was used. However, the CLASS variables were trial, stadium, and sample number; the MODEL statement included the log-transformed density of *P*. *agglomerans* Sc1R as the dependent (response) variable and the insect stadium as the independent variable. Trial and sample number were the RANDOM variables. Finally, the LSMEANS statement was invoked to generate mean log-transformed densities and the ADJUST = TUKEY option was used to generate the Tukey-Kramer Adjusted *P* statistic for comparison of means between stadia. To assess the log-transformed densities of general bacteria, the syntax was as described immediately above except that the log-transformed density of general bacteria was the dependent (response) variable. Also, methods for generating back-transformed means and range of back-transformed means were as described above for determination of similar data for *P*. *agglomerans* Sc1R.

## Results

### Frequency of *P*. *agglomerans* infection across stadia

Acquisition and retention of *P*. *agglomerans* Sc1R was observed across all stadia of southern green stink bug, from 1^st^ instars to adults (Table 1). In trial 1, a significantly higher number (i.e., all) of the 1^st^ instars possessed *P*. *agglomerans* Sc1R and fewer numbers of 3^rd^ instar possessed *P*. *agglomerans* Sc1R (Fisher’s Exact: *P* < 0.0001; Table 1) relative to the expected observations. Similarly, in pooled trials 2 and 3, significantly more than expected of 2^nd^ and 3^rd^ instars possessed *P*. *agglomerans* Sc1R and fewer than expected at 5^th^ instar and adults possessed *P*. *agglomerans* Sc1R (Fisher’s Exact: *P* < 0.0001; Table 1). In baseline (i.e., control) samples exposed to the sterile green beans, *P*. *agglomerans* Sc1R was not detected in any of the 1^st^ instars (trial 1) or 2^nd^ instars (pooled trials 2 and 3), confirming that our stock colony was free of the introduced pathogen.

**Table 1:** Frequency of *P*. *agglomerans* Sc1R infection and retention across stadia of southern green stink bug after initial exposure at 1^st^ or 2^nd^ instar.

Trial(s)	Stadium	*n*	% infection
1	1^st^ instar	20	100.00
	3^rd^ instar	20	5.00
	5^th^ instar	20	50.00
	Adult	21	71.43
2 & 3, pooled	2^nd^ instar	40	95.00
	3^rd^ instar	57	64.91
	5^th^ instar	56	21.43
	Adult	63	42.86

### Densities of *P*. *agglomerans* Sc1R across stadia

**Fig 1:**
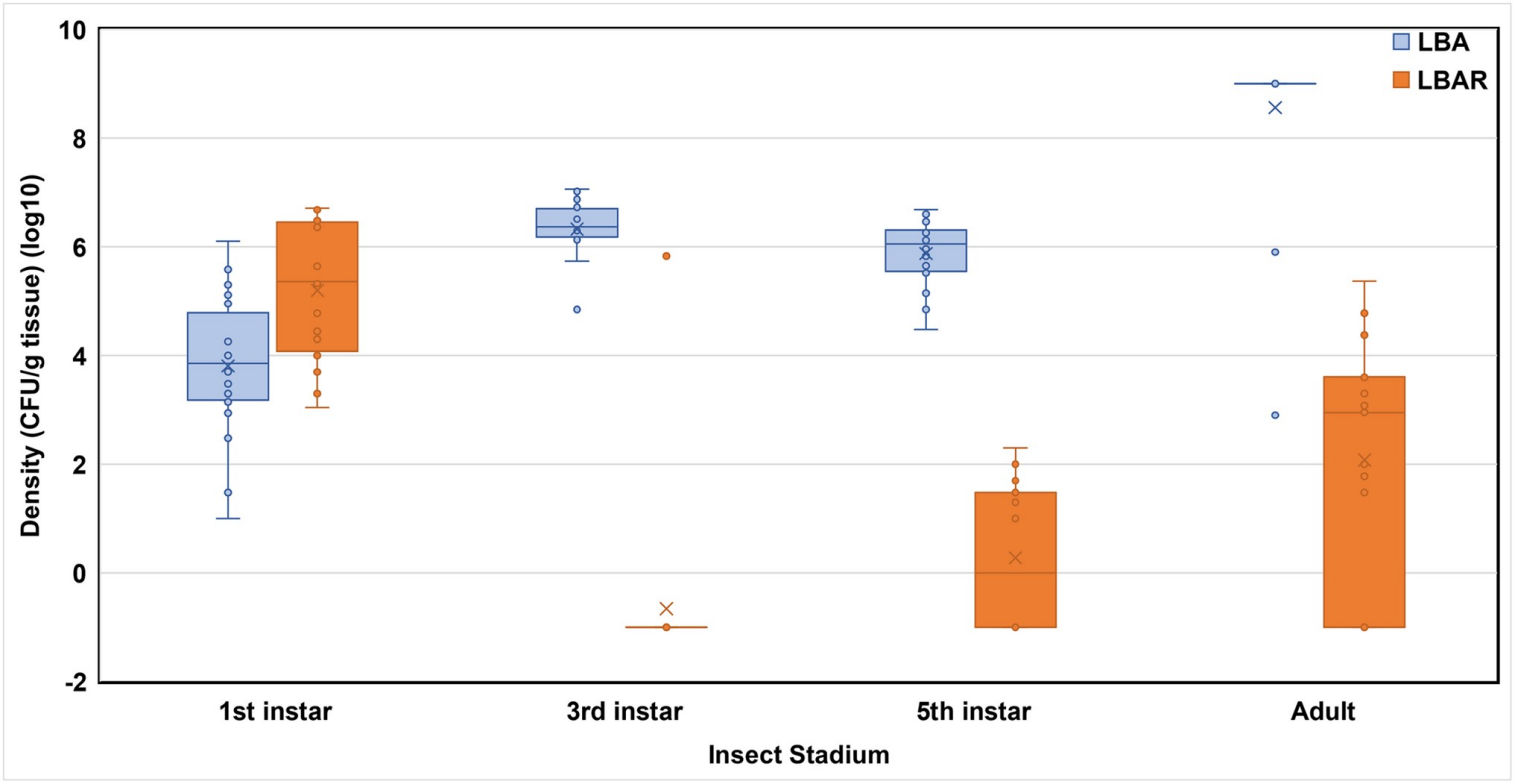
Raw log microbe densities (CFU/g tissue) across stadia of southern green stink bug, trial 1. LBA, densities of general bacteria propagated on Luria Bertani agar; LBAR, densities of *P*. *agglomerans* Sc1R on Luria Bertani agar amended with rifampicin. For each bar, × = mean; filled ○ = raw data point; line across bar = median.

**Fig 2:**
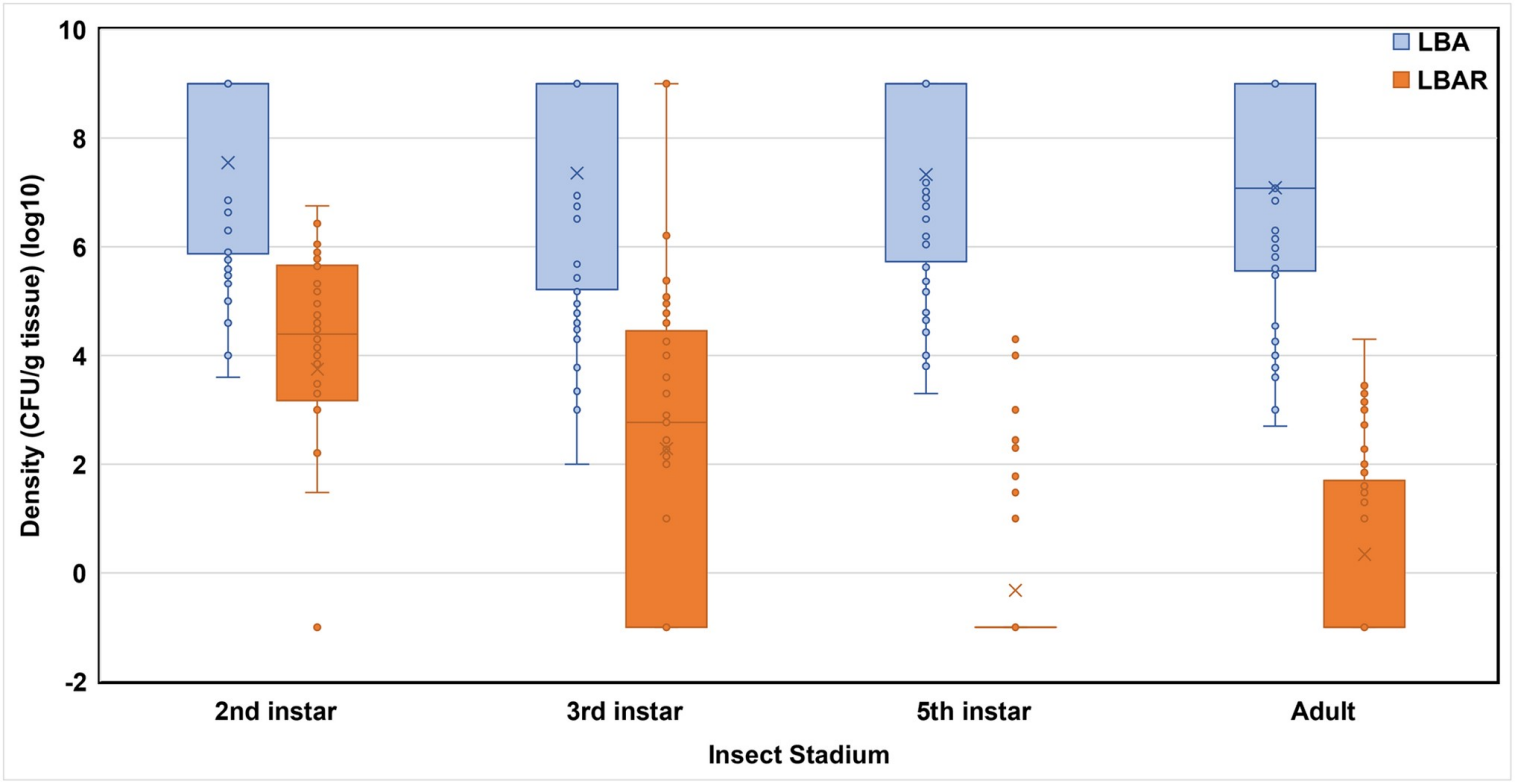
Raw log microbe densities (CFU/g tissue) across stadia of southern green stink bug, trials 2 & 3. LBA, densities of general bacteria propagated on Luria Bertani agar; LBAR, densities of *P*. *agglomerans* Sc1R on Luria Bertani agar amended with rifampicin. For each bar, × = mean; filled ○ = raw data point; line across bar = median.

*Pantoea agglomerans* Sc1R was ingested at 1^st^ and 2^nd^ instars and retained to adulthood (Table 2; Figs 1 and 2). Observed densities of *P*. *agglomerans* Sc1R were significantly affected by insect stadium in trial 1 (*F* = 49.78; df = 3, 38; *P* < 0.0001). The 1^st^ instars possessed significantly higher densities of *P*. *agglomerans* Sc1R than all other stadia, including adults (Adj. *P* < 0.0001 for all pairwise comparisons with 1^st^ instars; Table 2). The 3^rd^ and 5^th^ instars possessed significantly lower densities of *P*. *agglomerans* Sc1R and, although densities did not differ between these stadia (Adj. *P* = 0.27), densities in both stadia were significantly lower than those densities observed in adults (3^rd^ instar vs. adult, Adj. *P* < 0.0001; 5^th^ instar vs adult, Adj. *P* = 0.0048; Table 2).

**Table 2:** Log-transformed mean (LSMean ± SE) number of colony forming units (cfu/g tissue) of *P*. *agglomerans* Sc1R across various stadia of southern green stink bug after exposure of 1^st^ and 2^nd^ instars to *P*. *agglomerans* Sc1R.

Trial(s)	Stadium	*n*	Density (CFU/g tissue)	
			LSMean (± SE)	Back-transformed mean (range)
1	1^st^ instar	20	5.20a (0.36)	157,870.14 (68,155.36–365,678.98)
	3^rd^ instar	20	-0.66c (0.36)	0.22 (0.09–0.50)
	5^th^ instar	20	0.27c (0.36)	1.87 (0.81–4.34)
	Adult	21	2.08b (0.36)	119.48 (52.64–271.21)
2 & 3	2^nd^ instar	40	4.35a (0.33)	22,125.85 (10,360.00–47,249.80)
	3^rd^ instar	57	2.38b (0.27)	240.21 (128.71–448.33)
	5^th^ instar	56	-0.22c (0.27)	0.61 (0.32–1.14)
	Adult	63	0.37c (0.26)	2.37 (1.31–4.27)

Within Trials, log-transformed LSMeans followed by the same lowercase letter within a column are not significantly different. Means separation was based on Tukey’s Adjusted *P* values ≤ 0.05 derived from the ADJUST = TUKEY option in the LSMEANS statement.

Insect stadium also significantly influenced observed mean densities of *P*. *agglomerans* Sc1R across stadia in the pooled 2^nd^ and 3^rd^ trials (*F* = 49.56; df = 3, 146; *P* < 0.0001). Similar to trial 1, highest density of *P*. *agglomerans* Sc1R was observed at stadium of first exposure (i.e., 2^nd^ instar) and 2^nd^ instars possessed significantly higher densities than all other stadia, including adults (Adj. *P* < 0.0001 for all pairwise comparisons with 2^nd^ instars; Table 2). Overall, densities of *P*.*agglomerans* Sc1R declined as stadia matured with 5^th^ instars and adults having similar densities (Adj. *P* = 0.33) that were significantly lower than densities observed for 3^rd^ instars (Adj. *P* < 0.0001) and 2^nd^ instars (Adj. *P* < 0.0001) (Table 2). *Pantoea agglomerans* Sc1R was not detected in the control samples.

### Densities of normal microbiota across stadia

In trial 1, insect stadium significantly affected observed mean densities of general bacteria (*F* = 68.38; df = 3, 38; *p* < 0.0001). Densities of general bacteria in adults were significantly higher than all other stadia (Adj. *P* < 0.0001 for all pairwise comparisons with adults) (Table 3). Lowest densities were observed in 1^st^ instars and these were significantly lower than densities observed in 3^rd^ and 5^th^ instars (Adj. *P* < 0.0001 for all pairwise comparisons with 3^rd^ and 5^th^ instars) (Table 3). Observed densities between 3^rd^ and 5^th^ instars did not differ (Adj *P* = 0.52).

**Table 3:** Log-transformed mean (LSMean ± SE) number of colony forming units (cfu/g tissue) of general innate bacteria across various stadia of southern green stink bug after exposure of 1^st^ and 2^nd^ instars to *P*. *agglomerans* Sc1R.

Trial(s)	Stadium	*n*	Density (CFU/g tissue)	
			LSMean (± SE)	Back-transformed mean (range)
1	1^st^ instar	20	3.81c (0.24)	6,415.05 (3,692.33–11,145.51)
	3^rd^ instar	20	6.33b (0.24)	2,137,962.09 (1,230,552.08–3,714,496.90)
	5^th^ instar	20	5.88b (0.24)	759,626.31 (437,219.98–1,319,775.31)
	Adult	21	8.56a (0.23)	364,921,961.00 (212,813,904.60–625,748,763.40)
2 & 3	2^nd^ instar	40	7.80a (0.87)	62,704,686.37 (8,560,517.12–459,303,759.30)
	3^rd^ instar	57	7.11a (0.85)	13,100,865.86 (1,847,140.80–92,918,031.38)
	5^th^ instar	56	7.10a (0.85)	12,685,278.31 (1,786,487.58–90,074,114.22)
	Adult	63	7.48a (0.85)	30,401,849.17 (4,304,274.90–214,733,597.50)

Within Trials, log-transformed LSMeans followed by the same lowercase letter within a column are not significantly different. Means separation was based on Tukey’s Adjusted *P* values ≤ 0.05 derived from the ADJUST = TUKEY option in the LSMEANS statement.

Insect stadium did not significantly affect observed mean densities of innate microbiota in the pooled trials 2 and 3 (*F* = 1.45; df = 3, 146; *P* = 0.23). Mean densities were not significantly different across stadia (0.2924 < Adj. *P* < 1.000) (Table 3).

Time of initial exposure to *P*. *agglomerans* Sc1R seemingly affected the densities of observed general bacteria and *P*. *agglomerans* Sc1R (Figs 1 & 2). Overall, when 1^st^ instars were exposed to *P*. *agglomerans* Sc1R, the densities of general bacteria were initially lower than densities of *P*. *agglomerans* Sc1R but densities of general bacteria increased in later stadia (Fig 1).

## Discussion

Our data provide definitive evidence that southern green stink bugs can acquire *P*. *agglomerans* Sc1R during the 1^st^ and 2^nd^ instars and can retain the pathogen across multiple subsequent stadia to adulthood. Confirmed acquisition of *P*. *agglomerans* Sc1R by 1^st^ instars further support earlier findings [9] which demonstrated that 1^st^ instars do feed, contrary to previous dogma. Retention of the pathogen to subsequent instars also support the earlier report of *P*. *agglomerans* Sc1R in 2^nd^ instars after 1^st^ instars completed the molting process [9]. Similarly, pathogen retention between 5^th^ instars and adults was observed when 5^th^ instars of *Chinavia hilaris* Say [reported as *Acrosternum hilare* (Say)] infected with *E*. *coryli* (reported as *N*. *coryli*) molted to adults and *E*. *coryli* was detected in adults [8].

Densities of *P*. *agglomerans* Sc1R varied over stadia (Figs 1 & 2) and densities were highest at 1^st^ (trial 1; Fig 1) and 2^nd^ instar (trials 2 and 3; Fig 2) and generally declined across subsequent stadia. Lower densities in the 3^rd^ and 5^th^ instars may have been a function of the lower frequency of infected individuals at these stages in trial 1 (Table 1). Conversely, the higher densities observed at 1^st^ instars and adults were likely influenced by the high frequencies of infection at these stages (Table 1). Similarly, in trial 2, lower densities in 5^th^ instars and adults could presumably be attributed to the lower rate of infection in 5^th^ instars and adults (Table 1).

Densities of innate microbiota varied over stadia but, unlike densities of *P*. *agglomerans* Sc1R, the mean densities of innate microbiota increased over time (trial 1) or remained static over all stadia (pooled trials 2 & 3; Fig 2). In pooled trials 2 &3, Densities were numerically higher in 2^nd^ instars followed by adults, 3^rd^ instars, and 5^th^ instars. Interestingly, the pattern of higher densities in 3^rd^ instars vs 5^th^ instars was also observed in trial 1. Lower densities of general bacteria, especially at 1^st^ instar, could potentially be due to a lack of innate bacteria establishing during this stadium because of exposing newly hatched nymphs to treated beans. Newly hatched nymphs typically congregate atop or about the source egg clutch to probe and acquire endosymbionts [12]. Conversely, when 1^st^ instars could feed freely on provisioned green beans before exposure to treated beans at 2^nd^ instars (i.e., during pooled trials 2 &3), the densities of general bacteria were the highest detected throughout the study and these densities continued throughout all subsequent stadia (Fig 2). However, determining the influence of time of exposure was outside the scope of this study but these general observations suggest additional research may be warranted to further elucidate these dynamics. Nonetheless, southern green stink bug can retain *P*. *agglomerans* Sc1R to adulthood if ingested at earlier stadia.

The digestive tract of the southern green stink bug is partitioned into four distinct sections [13–15], and each section has differing and specific physiological functions [16]. Because resection of specific midgut anatomy for microbiological processing was not conducted in the current study, it is unknown whether *P*. *agglomerans* Sc1R was in each of the midgut sections or whether colonization in a specific midgut section affected densities of *P*. *agglomerans* Sc1R. Regardless, the detected retention of *P*. *agglomerans* Sc1R across stadia could be due to the physiological processes during molting. Specifically, during each molt, “[a]ll the cuticular parts are shed, including the intima of fore- and hind-gut, the endophragmal skeleton and the linings of the tracheae…" and that "[t]he midgut does not have a cuticular lining, but in the majority of insects it is lined by a delicate peritrophic membrane" [17]. Thus, in the current study, *P*. *agglomerans* Sc1R (and innate microbiota) cells evidently were residing in the midgut of the various stadia. Although, previous work showed that, in adult *N*. *viridula*, *P*. *agglomerans* Sc1R can also colonize in the rostrum and the head [10].

Pathogen infection frequency can be affected by infected insects depositing pathogens on a food source or pathogen acquisition from the environment [18, 19]. Further, a plant pathogen can remain virulent in fecal matter after passing through the alimentary system of *C*. *hilaris* [8]. Because stink bugs have been observed probing fecal deposits (JFE, pers. obs.), Petri plates in the current study were replaced at each molt to exclude or mitigate acquisition from fecal deposits as a variable. However, the persistence, prevalence, and virulence of *P*. *agglomerans* Sc1R in fecal matter of *N*. *viridula* has yet to be determined. Therefore, assessing the presence and virulence of *P*. *agglomerans* Sc1R in the feces of *N*. *viridula* (and pathogen acquisition rates by insects from fecal matter) remains a key research avenue that is currently being addressed.

In addition to presence and concentrations, densities of *P*. *agglomerans* Sc1R within infected stadia of *N*. *viridula* require additional evaluation. The current study sheds new light on the variation of temporal densities among stadia, but data are lacking regarding the pathogen infective concentration required to cause the total loss (i.e., infection and necrosis of lint) of a cotton boll or other high-value crop, since *N*. *viridula* is an extremely polyphagous pest [1]. For example, what is a minimum infective pathogen concentration (i.e., would a stink bug harboring 10 cfus cause infection and total loss or are 10^9^ cfus required to cause similar damage)? Differing pathogens have differing infective doses [20], but similar work is lacking to differentiate infective cell numbers for *P*. *agglomerans* Sc1R. Also, does pathogen quorum sensing [21, 22] correlate with disease severity? Finally, Figs 1 and 2 indicate a negative correlation exists with a reduced number of *P*. *agglomerans* Sc1R densities but higher general bacteria microbiota densities at later instars. This pattern could presumably be due to: a function of reduced sample sizes of insects exposed to *P*. *agglomerans* Sc1R; the single ‘inoculation’ (i.e., feeding bout) with *P*. *agglomerans* Sc1R (versus persistent exposure to the pathogen at all stadia); persistence, prevalence, and virulence of *P*. *agglomerans* Sc1R after initial inoculation versus innate general bacteria; or, a combination thereof.

In combination with earlier reports on stink bug stylet penetration potential [23, 24], our current findings suggest various insect stadia of *N*. *viridula* could contribute to pathogen transmission. Adult *N*. *viridula* have been used as the study organism in all studies documenting transmission of *P*. *agglomerans* Sc1R [2, 3]. However, given that densities of *P*. *agglomerans* Sc1R in nymphs have been quantified in the current study, can nymphs transmit *P*. *agglomerans* Sc1R? This question is validated by earlier findings related to stylet penetration and boll wall thickness [23, 24]. Specifically, stylet penetration estimates for late instars and adults of *N*. *viridula* [23] (and other adult pentatomids [25]) were determined and the estimates for late instars indicate possible breaching of the cotton boll wall as early as 1-d after flower [24]. The susceptibility of bolls at 1-d after flower potentially allows for the introduction of plant pathogens into cotton bolls by late instars of *N*. *viridula*. Thus, examining the potential of transmission by nymphs would provide a more comprehensive understanding of the southern green stink bug / pathogen / plant complex.
